# Early Weight-Based Aggressive vs. Non-Aggressive Goal-Directed Fluid Resuscitation in the Early Phase of Acute Pancreatitis: An Open-Label Multicenter Randomized Controlled Trial (The WATERFALL Trial), Design, and Rationale

**DOI:** 10.3389/fmed.2020.00440

**Published:** 2020-09-02

**Authors:** Federico Bolado, James L. Buxbaum, Alicia Vaillo-Rocamora, Karina Cárdenas-Jaén, Patrick Maisonneuve, Enrique de-Madaria

**Affiliations:** ^1^Gastroenterology Department, Complejo Hospitalario de Navarra, Pamplona, Spain; ^2^Division of Gastrointestinal and Liver Diseases, Keck School of Medicine of University of Southern California, Los Angeles, CA, United States; ^3^Gastroenterology Department, Alicante University General Hospital, Alicante Institute for Health and Biomedical Research (ISABIAL), Alicante, Spain; ^4^Division of Epidemiology and Biostatistics, IEO-Istituto Europeo di Oncologia IRCCS, Milan, Italy

**Keywords:** fluid resuscitation, ringer lactate, fluid therapy, acute pancreatitis (AP), randomized controlled (clinical) trial

## Abstract

Treatment options are limited for acute pancreatitis (AP). Early aggressive fluid resuscitation (AFR) has been widely considered beneficial because of theoretical improvement in end-organ perfusion, including the pancreas and gut, with pancreatic necrosis and bacterial translocation as consequences of ischemia. There is scarce direct evidence for its association to improved outcomes. Furthermore, it has been described that AFR may be associated with poor outcomes in severe AP. WATERFALL is an investigator-initiated international multicenter open-label randomized controlled trial comparing AFR vs. moderate fluid resuscitation (MFR) in AP. The main outcome variable will be the incidence of moderate to severe AP (a clinically relevant outcome that has been validated). Aggressive fluid resuscitation will consist in lactated Ringer solution (LR) 20-mL/kg bolus (administered over 2 h) followed by LR 3 mL/kg per hour. Patients randomized to MFR will receive an LR bolus 10 mL/kg in case of hypovolemia or no bolus in patients with normal volemia, followed by LR 1.5 mL/kg per hour. The patients will be assessed at 3 (±1), 12 (±4), 24 (±4), 48 (±4), and 72 (±4) h from recruitment, and fluid resuscitation will be adjusted to the patient's clinical and analytical status according to a protocol. Based on a prospective multicenter study, the incidence of moderate to severe AP is 35%. Sample sizes of 372 patients per group (overall 744) achieve 80% power to detect a difference in the incidence of moderate to severe AP of 10%, at a significance level (α) of 0.05 using a two-sided *z*-test, assuming a 10% dropout rate. These results assume that three sequential tests are made using the O'Brien–Fleming spending function to determine the test boundaries.

## Introduction

Acute pancreatitis (AP) is the third leading cause of hospital admission for gastrointestinal disease (1). While the majority of patients with AP have a mild course, 35% develop moderate to severe disease, which is associated with high morbidity and an increased risk of mortality (2). Thus, a vital aim in the early management of AP is to decrease the incidence of moderate to severe disease. Unfortunately, there are currently no specific therapies for AP, so the cornerstone in the management of this frequent disease is supportive treatment, including fluid resuscitation, analgesia, and close monitoring for organ failure (3).

Since late 1990s, experts have recommended aggressive fluid resuscitation (AFR) in AP (4) based on an observed correlation between hemoconcentration and necrosis (5). Aggressive fluid resuscitation became a dogma in pancreatology, but it was based on retrospective studies at high risk of biases (6). In 2011, a prospective cohort study suggested that AFR was associated with poor outcomes in AP (7). In 2017, an international multicenter observational study of more than 1,000 patients reported that there was not a clear correlation between early AFR and improved outcomes (8).

Randomized controlled trials (RCTs) of fluid resuscitation for AP have been limited by small sample sizes and flawed design. Two small RCTs from the same group from China described that patients with severe AP had unfavorable outcomes including higher mortality rate in the context of AFR (9, 10). Another study by Buxbaum et al. (11) in the United States suggested that AFR hastens clinical improvement among patients with predicted mild AP, but was not powered to address clinically important outcomes, such as the development of organ failure (12). Moderate to severe AP as defined by the revised Atlanta classification (13) has been validated as a clinically relevant outcome variable in several studies, including our nationwide Spanish multicenter prospective cohort study involving more than 1,600 patients (2).

Few RCTs on AP have taken into account patient symptoms. PAN-PROMISE is a recently validated patient-reported outcome measurement scale for AP (14), thus making possible to know the impact of this disease on patients' wellness.

An adequately powered RCT focused on clinically relevant outcome variables and taking into account the patients' perspective is needed to define the appropriate fluid strategy in AP.

## Methods and Analysis

### Design

**Figure 1:**
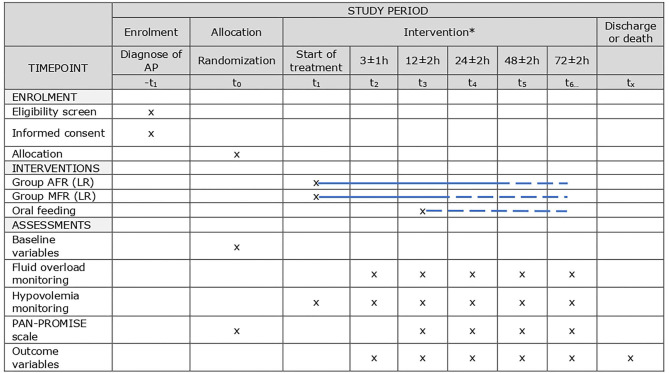
SPIRIT scheduled enrollment, interventions, and assessments. *The detailed protocol is illustrated in Figure 2. LR, lactated Ringer. Dashed lines stands for optional, according to patient status.

WATERFALL is an investigator-initiated international multicenter open-label RCT comparing early AFR vs. moderate fluid resuscitation (MFR). The study is endorsed by the Spanish Association of Pancreatology (AESPANC) and the Spanish Association of Gastroenterology (AEG). This trial protocol follows the Standard protocol items: recommendations for interventional trials (SPIRIT) guidelines (15) (Figure 1).

### Population

Consecutive patients with clinical suspicion of AP in the emergency room of any of the collaborating centers (Supplementary Material 1) will be evaluated to participate in the study.

### Inclusion Criteria

(1) Patients 18 years or older

(2) Diagnosis of AP according to the revised Atlanta classification (13), which requires two of the following three criteria: (A) typical abdominal pain, (B) increase in serum amylase or lipase levels higher than three times the upper limit of normality, and (C) signs of AP in imaging.

### Exclusion Criteria

Patients will be excluded if they fulfill any of the following criteria:

1. Uncontrolled arterial hypertension (systolic blood pressure >180 and/or diastolic blood pressure >100 mmHg)
2. New York Heart Association class II heart failure (slight limitation of physical activity; fatigue, palpitations, or dyspnea with ordinal physical activity) or worse, or ejection fraction <50% in the last echocardiography
3. Decompensated cirrhosis (Child's class B or C)
4. Hyper or hyponatremia (<135 or >145 mEq/L)
5. Hyperkalemia (>5 mEq/L)
6. Hypercalcemia (albumin or protein-corrected calcium >10.5 mg/dL)
7. Baseline kidney failure (basal glomerular filtration rate <60 mL/min per 1.73 m^2^)
8. Clinical signs or symptoms of volume overload or heart failure at recruitment (dyspnea, peripheral edema, pulmonary rales, or evident increased jugular ingurgitation at 45°)
9. Shock or respiratory failure according to the revised Atlanta classification at recruitment (non-fluid-responding systolic blood pressure <90 mmHg, Pao_2_/Fio_2_ ≤ 300)
10. Time from pain onset to arrival to emergency room >24 h
11. Time from confirmation of pancreatitis to randomization >8 h
12. Severe comorbidity associated with an estimated life expectancy <1 year
13. Confirmed chronic pancreatitis [in case of recurrent alcoholic pancreatitis a recent (<6 months) computed tomography (CT) scan/magnetic resonance imaging (MRI) or endoscopic ultrasound is needed to rule out chronic pancreatitis]

### Recruitment, Randomization, and Data Acquisition

Recruitment will be performed by collaborating gastroenterologists and/or surgeons of the participating centers.

Patients who meet the eligibility criteria will be randomly assigned to AFR or MFR after informed consent. The electronic case report form (eCRD) will be based on RedCAP web-based application (16) (AEG node). Randomization will be performed automatically by REDCap, stratified by center, presence of systemic inflammatory response syndrome (SIRS), and suspected baseline hypovolemia (see below).

### Treatment Protocol

1. Definitions
  Fluid overload:
  Fluid overload is defined by the presence of at least two of the following three criteria (adapted from Sharma et al. definition of heart failure) (17):
  Criteria 1. Hemodynamic-imaging evidence (≥1):
  - Non-invasive diagnostic evidence of heart failure [i.e., echocardiographic, cardiac (MRI)]
  - Radiographic evidence of pulmonary congestion
  - Invasive cardiac catheterization suggesting evidence of heart failure [i.e., pulmonary capillary wedge pressure (or left ventricular end-diastolic pressure) >18 mmHg, right arterial pressure [or central venous pressure] >12 mmHg, or cardiac index <2.2 L/min per m^2^]

Criteria 2. Heart failure symptoms (1):

- Dyspnea

Criteria 3. Heart failure signs (≥1):

- Peripheral edema
- Pulmonary rales or crackles, or crepitation
- Increased jugular venous pressure, hepatojugular reflux, or both

Additionally, in those with suspected fluid overload, acute respiratory distress syndrome (ARDS) must be ruled out. Exclusion of ARDS for this may be met by one of two criteria:

1. Prompt response to diuretics and/or decrease in fluid resuscitation volume rate and/or hemodialysis-hemofiltration
2. Absence of ARDS criterion (for ARDS, the patient must meet all the following four criteria as defined by the modified Berlin definition, ARDS Definition Task Force, JAMA 2012)
  (A) Onset within 1 week of the pancreatitis
  (B) Bilateral opacities not fully explained by effusions, lobar collapse, or nodules
  (C) Respiratory failure not fully explained by cardiac failure or fluid overload needs objective assessment (i.e., echocardiography) to exclude hydrostatic edema if no risk factor is present
  (D) Pao_2_/Fio_2_ ≤ 300

Severity of fluid overload will be classified into three categories:

- Mild: Patients respond to medical treatment or decrease in volume infusion rate, and the Pao_2_/Fio_2_ never decreases <300.
- Moderate: Patients respond to medical treatment or decrease in volume infusion rate and have at least one measurement with Pao_2_/Fio_2_ <300.
- Severe: Patients require invasive or non-invasive mechanical ventilation, and/or hemofiltration, or expire due to overload. It is crucial to rule out ARDS in this scenario (see above).

Hypovolemia:

Hypovolemia is defined by the presence of one criterion or more:

1. Baseline creatinine >1.1 mg/dL or blood urea nitrogen (BUN) >20 mg/dl, equivalent to urea >43 mg/dL
2. Hematocrit >44%
3. Increase in creatinine and/or BUN and/or urea from the previous value
4. Urine output <0.75 mL/kg per hour
5. Systolic blood pressure <90 mmHg without other explanation than hypovolemia
6. Signs and/or symptoms of dehydration (intense thirst, dehydrated oral mucosa, decreased skin turgor–skin pinch

Systemic inflammatory response syndrome:

Systemic inflammatory response syndrome will be defined by the presence of two or more of the following criteria:

1. Leukocyte count <4,000 or >12,000/mm^3^
2. Heart rate >90/min
3. Respiratory rate >20 breaths/min or Pco_2_ <32 mmHg
4. Temperature (Celsius) <36 or >38°C

Criteria to start oral feeding

Feeding “per os” will be initiated when:

(A) The intensity of abdominal pain is <5 over 10 (0 = absence of pain and 10 = maximum possible pain); and
(B) The patient feels that he/she can tolerate oral feeding

2. Treatment Arms

All patients included in the study will be randomly assigned to group AFR or MFR.

#### Group AFR (See Flowchart in Figure 2)

Patients randomized to AFR will receive a 20-mL/kg bolus of lactated Ringer solution (LR) administered over 2 h, followed by an infusion at 3 mL/kg per hour. Fluid overload will be ruled out at 3 ± 1 h after randomization. Afterward, there are four checkpoints: 12 (±4), 24 (±4), 48 (±4), and 72 (±4) h after randomization. On each of them, criteria for hypovolemia, fluid overload, and for oral feeding are checked. According to the patient status:

(A) If no fluid overload or hypovolemia criteria are met, the LR infusion rate will be reduced to 1.5 mL/kg per hour.
(B) If criteria for fluid overload but no hypovolemia are met, the infusion rate of LR will be decreased or stopped, and if needed, the study physicians will consider diuretics and/or O_2_ as well as electrocardiogram chest X-ray and blood gases according to their clinical judgment. In case of refractory signs/symptoms of fluid overload, intensive care unit (ICU) assessment will be obtained.
(C) If criteria for hypovolemia without fluid overload are met, a bolus of LR 20 mL/kg over 2 h will be given followed by an infusion of LR 3 mL/kg per hour. One or more additional 20-mL/kg boluses may be given prior to the 24-h checkpoint only in case of urine output <0.5 mL/kg per hour or hypotension (systolic blood pressure <90 mmHg). In case of refractory hypotension, ICU assessment will be obtained.
(D) If fluid overload and hypovolemia criteria are both met, management should be performed according to the physician clinical judgment; in difficult cases, ICU assessment will be obtained.

Fluid resuscitation will be stopped at 48 h after randomization or later in patients without hypovolemia, tolerating oral feeding for at least 8 h. Lactated Ringer solution infusion should be maintained in case of hypovolemia or intolerance to oral feeding. Recommendations for enteral nutrition are explained bellow (general management).

**Figure 2:**
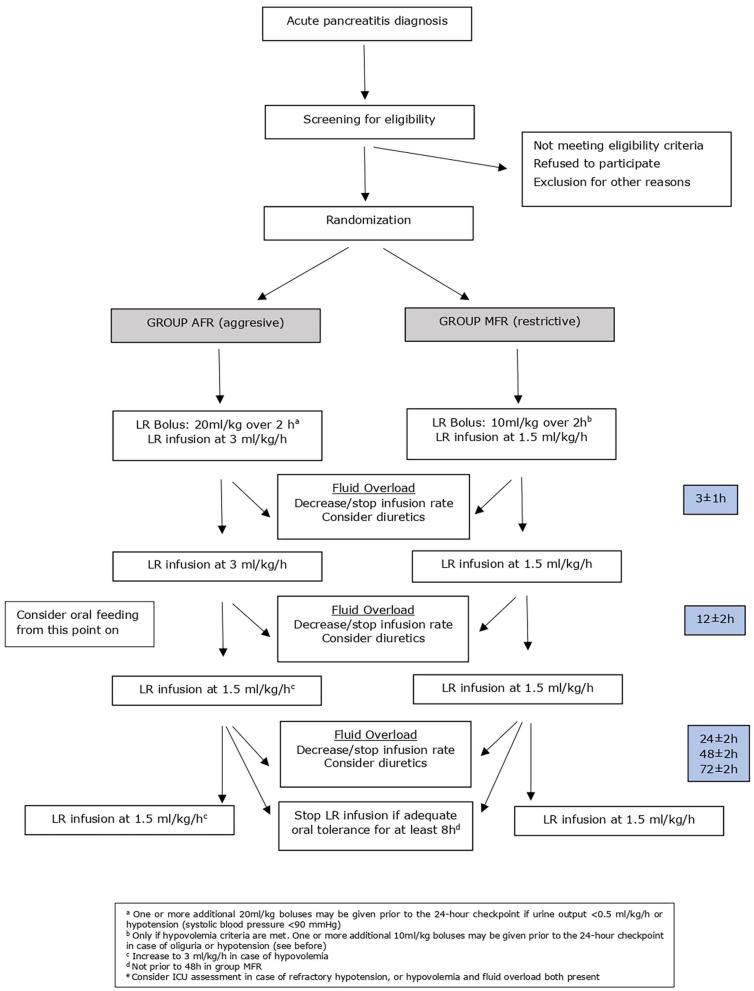
Detailed treatment protocol.

#### Group MFR (See Flowchart in Figure 2)

Group MFR will receive an LR infusion at 1.5 mL/kg per hour. A prior LR bolus of 10 mL/kg over 2 h should be administered only if criteria for hypovolemia are found. Fluid overload will be ruled out at 3 ± 1 h after starting the study treatment. Afterward, following the same checkpoints than in group AFR, patient management is as follows:

(A) If no fluid overload or hypovolemia criteria are met, LR infusion will be continued at 1.5 mL/kg per hour and criteria to start oral feeding will be assessed. After 8 h tolerating oral feeding, the LR infusion can be stopped.
(B) If fluid overload but not hypovolemia criteria are met, the infusion rate of LR will be decreased or stopped, and if needed, the study physicians will consider diuretics and/or O_2_, as well as electrocardiogram chest X-ray and blood gases if necessary according to their clinical judgment. Patients will be evaluated for criteria to start oral feeding. In case of refractory signs/symptoms of fluid overload, ICU assessment will be obtained.
(C) If criteria for hypovolemia but not fluid overload are met, a bolus of LR 10 mL/kg over 2 h will be given followed by infusion of LR 1.5 mL/kg per hour. One or more additional 10-mL/kg boluses may be given prior to the 24-h checkpoint only in case of urine output <0.5 mL/kg per hour or hypotension (systolic blood pressure <90 mmHg). In case of refractory hypotension, ICU assessment will be obtained.
(D) If fluid overload and hypovolemia criteria are both met, management should be performed according to the physician clinical judgment, in difficult cases ICU assessment will be obtained.

Thus, in the MFR group, fluid resuscitation can be stopped as early as 20 h after randomization, if the patient tolerates for 8 h, oral feeding started at 12 (±4)h.

3. General Management
  - Blood test (hematocrit, leukocyte count, BUN, urea, and creatinine) will be obtained at 12 h (±4), 24 h (±4), 48 h (±4), and 72 h (±4) in all patients.
  - A CT scan is recommended to be performed at least 72 h after recruitment to those patients with SIRS at emergency room, with persistent pain (>5 over 10) for more than 48 h, persistent intolerance to oral feeding, C-reactive protein >150 mg/L at 48 h, or in case of suspicion of local complications.
  - All patients must receive at least potassium 40 mEq/day unless it is contraindicated.
  - In case of diabetes, the use of insulin and dextrose solutions will be decided by the attending physician. In non-diabetic patients, dextrose use is discouraged.
  - Enteral nutrition can be administered according to the managing physician judgment. We recommend it in patients who do not tolerate oral feeding at 72 h from recruitment. Parenteral nutrition can be used in patients not tolerating oral or enteral feeding.

### Aims

This study aims to compare in patients with AP the effect of an aggressive vs. a MFR strategy on outcomes.

### Endpoints

**Main endpoint**: Our primary endpoint is to compare the impact of early and aggressive vs. a moderate, more restrictive fluid resuscitation on the incidence of moderate to severe AP according to the revision of the Atlanta classification (13). It includes patients with at least one of the following three criteria:

- Local complications (acute peripancreatic fluid collections or pancreatic necrosis or/and peripancreatic fat necrosis); or
- Exacerbation of previous comorbidity; or
- Organ failure (modified Marshall classification ≥2: creatinine ≥1.9 mg/dL and/or systolic blood pressure <90 mmHg despite fluid resuscitation and/or Pao_2_/Fio_2_ ≤ 300).

**Secondary aims**: Additional aims include a comparison of the following outcomes among the treatment arms:

(A) PAN-PROMISE scale (14) (Table 1) will be obtained at recruitment, and at 12 h (±4), 24 h (±4), 48 h (±4), and 72 h (±4) checkpoints
(B) Mortality
(C) Transient or persistent (>48 h) organ failure (cardiovascular, kidney, respiratory) (13).
(D) Local complications (13).
(E) Fluid overload
(F) Length of hospital stay
(G) ICU stay (admission or not, and length of stay)
(H) Need for invasive treatment
(I) Need for nutritional support
(J) Serum C-reactive protein at 48 and 72 h
(K) SIRS criteria at 12, 24, 48, and 72 h. Transient or persistent (>48 h) SIRS
(L) Combined variable: death and/or persistent organ failure and/or infection of pancreatic necrosis (18)

**Table 1:** PAN-PROMISE scale (14).

Each item is scored from 0 to 10. The patient should be asked for the worst score in the last 24 h (0 = none, 10 = the highest possible intensity)
A. Pain, especially in the abdomen, chest, or back B. Abdominal distention (bloating, sensation of excess gas) C. Difficulty eating, sensation of food being stuck in the stomach D. Difficulty with bowel movements (constipation or straining on bowel movements) E. Nausea and/or vomiting F. Thirst G. Weakness, lack of energy, fatigue, difficulty moving

### Sample Size

The sample size was calculated based on the main endpoint. Our prior multicenter study indicated a baseline incidence of moderate to severe AP of 35% (2). Sample sizes of 372 in each group achieve 80% power to detect a difference of 10% reduction between the group incidence of moderate to severe disease (from 35 to 25%) at a significance level (α) of 0.05, using a two-sided *z* test. We anticipated a dropout rate of 10%. These results assume that three sequential tests are made using the O'Brien–Fleming spending function to determine the test boundaries.

### Data Analysis

All analyses will be performed on an intention-to-treat basis.

The O'Brien–Fleming test, a multiple-testing procedure using group-sequential design for two proportions, will be used for comparing both treatments. The three sequential tests are two interim analyses and the final one. Accordingly, the trial could be stopped early for efficacy (primary endpoint) if the observed two-sided *P*-value is <0.0002 at the first interim analysis (after one-third of patients have been enrolled) or is <0.012 at second interim analysis (after two-thirds of patients have been enrolled), favoring AFR. At final analysis, the hypothesis that the incidence of moderate to severe pancreatitis is similar in the two treatment arms will be rejected if *P* <0.046. Estimates were calculated with the PASS 2008 software (NCSS, LLC. Kaysville, UT, USA).

Descriptive analysis will be expressed in mean (standard deviation), median (interquartile range), or *n* (%). Normality will be assessed by means of the Shapiro–Wilk test. Differences in continuous variables between the treatment arms will be compared by Student *t*-test or Mann–Whitney *U* test. Categorical variables will be compared using χ^2^ test (with Fisher correction when needed). Comparison of secondary endpoints will be expressed in terms of a relative risk and corresponding 95% confidence intervals. In case of statistically significant differences in baseline characteristics, a multivariable logistic regression analysis will be performed to correct it. A two-sided *P*-value of <0.05 will be considered statistically significant. Calculation will be performed with SPSS 21.0 (IBM, Armonk, NY, USA).

Predefined subgroup analysis will be performed in patients with and without SIRS at admission, persistent (>48 h) SIRS, and hypovolemia at admission.

The report of the results will follow the CONSORT (Consolidated Standards of Reporting Trials) statement (19).

### Data and Safety Monitoring Board

The Data and Safety Monitoring Board (DSMB) is an independent expert committee in charge of monitoring the data to guarantee safety of both recruited patients and patients to be recruited. An initial meeting will take place at the beginning of recruitment to plan scheduled future meetings. The DSMB will have access to updated anonymized data stored on the electronic case report form. The DSMB can advise to stop the study, in case of clear evidence of efficacy or harm in one treatment arm over the other, or in case of a slow recruitment rate.

Data and Safety Monitoring Board members are P. Zapater [Department of Clinical Pharmacology, Alicante University General Hospital (AUGH) with experience in clinical trials, statistics, and drug safety], R. Jover (Department of Gastroenterology, AUGH, with experience in clinical trials in the gastroenterology field), and V. Climent (Department of Cardiology, AUGH, an expert on heart failure and fluid overload).

### Study Duration

The anticipated study duration is 3 years. The DSMB may advise to halt the study previously due to safety issues or clear evidence for a more effective treatment arm (as explained in Sample Size, three analyses will be performed).

## Discussion

Acute pancreatitis is a frequent cause of admission, which entails a significant economic burden (1). Some advances have been made in recent years in understanding the pathophysiology and severity determinants of this disease, but it still lacks a specific treatment. Observational studies showed a close relationship between hemoconcentration and necrosis and hypothesized that AFR may prevent pancreatic necrosis by increasing pancreatic blood flow (5). Furthermore, correcting hypovolemia may be important for other end organs. Persisting splanchnic vasoconstriction in response to AP and hypovolemia secondary to third space fluid loss may be associated with ischemic injury to the gut and thus to increased intestinal permeability, which may lead to bacterial translocation and a subsequent SIRS (20). However, there are no RCTs showing a direct relationship between AFR and a decrease in local complications. The available RCT showing benefits for AFR included only predicted mild AP and used in fact intermediate or surrogate endpoints (11), as more robust ones are not feasible for a single-center study. The only RCTs that used robust outcomes were performed on severe AP and described a deleterious effect of AFR on those very sick patients (9, 10). WATERFALL aims to compare an aggressive vs. a restrictive fluid resuscitation strategy. The study has been designed to detect a decrease on the incidence of complications in AP and will monitor carefully the incidence of adverse effects of AFR (fluid overload). Serious concerns have emerged about the safety of high-dose fluids (7), especially in patients with comorbidities and elderly patients. WATERFALL has no age limit for enrolment. Acute pancreatitis incidence increases with age, so excluding older people would result in a decreased external validity. A close evaluation of fluid overload signs and symptoms will be carried out throughout the study period. As stated above, an independent expert committee will monitor patients' safety and might advise to stop the study if the harms of an arm clearly exceed those of the other.

One important aspect of WATERFALL will be to explore the effect of fluid resuscitation on the patient's symptoms. We may hypothesize that AFR may be associated with decreased thirst [an important symptom for patients with AP (14)] but may increase abdominal distension or even decrease oral tolerance of food. For these reasons, changes in fluid policy may result in changes in patients' wellness. Our study will also focus on this frequently eluded point, using the PAN-PROMISE scale, a specific patient-reported outcome measurement for AP (14).

Fluid therapy will be based on LR based on its anti-inflammatory effect (21).

WATERFALL aims to include patients with different severity and to have a validated clinically relevant endpoint. Several studies (2, 22), including a prospective nationwide study (2), have shown that the different categories of severity of the revised Atlanta classification are associated with different important outcomes, including hospital stay and mortality.

In conclusion, WATERFALL aims to answer some vital clinical questions in AP: does early and AFR with LR improve relevant endpoints? Is it safe in all patients? Fluid resuscitation is a widely available, inexpensive therapy. Therefore, the demonstration of a positive or negative effect of AFR will result in immediate and important changes in clinical practice.

## Ethics Statement

The study was originally approved on May 29th 2019 by Alicante University General Hospital (AUGH) Institutional Review Board (Comité Ético de Investigación con Medicamentos del Hospital General Universitario de Alicante, CEIM HGUA, reference number 2019/003).

Each patient will be informed of the aims, methods, and possible consequences (both potential benefits and harms) of the study. Participation in this study requires signed informed consent. The patients will be able to withdraw from the study at any time. Researchers will emphasize that refusing to be included or withdrawing from the study will have no consequences on their management or rights.

Data acquisition, management, and use will be in accordance with the European Union regulation 2016/679, European Parliament and Council, April 27, 2016. Each subject will be assigned a code, and all data will be recorded using that number. Only the attending physicians will know the relationship between the code and patient's identity. Access to personal information will be restricted to the study physician and collaborators, health authorities, and DSMB members, only if needed for safety issues, to review study data and proceedings, but always maintaining patients' confidentiality.

All data generated during this study are intended to be published. The WATERFALL trial has been registered in ClinicalTrials.gov (number pending).

General rules for authorship are explained in Table 2.

**Table 2:** Rules for authorship and access to the database.

**Recruited patients**	**Rights**
**Collaborators will be considered as authors depending of the number of patients recruited without missing data:**	
<6% of the overall number of patients recruited, <42 patients (<14 patients/year) if the study is finished at 700 patients	Access to data from the same center One author in collateral studies, no author in the main study
6–10% patients or 42 to 70 if the study is finished at 700 patients	Access to data from the same center One author in the main study
>10–15% patients, >70–105 if the study is finished at 700 patients	Access to data from the same center Two authors in the main study Can apply to access to whole database for *post-hoc* studies
>15% patients, >105 if the study is finished at 700 patients	Access to data from the same center Three authors in the main study Can apply to access to whole database for *post-hoc* studies

*Please note that the scientific journal may limit the number of authors, in that case we will include as many authors as possible based on the number of patients recruited*.

## Author Contributions

Ed-M was the trial sponsor and PI. The study was designed by Ed-M, FB, and JB. PM reviewed the statistics. AV-R and KC-J critically assessed the study design. All authors edited the manuscript, and read and approved the final manuscript.

## Conflict of Interest

The authors declare that the research was conducted in the absence of any commercial or financial relationships that could be construed as a potential conflict of interest.

## Abbreviations

AEG: Spanish Association of Gastroenterology
AESPANC: Spanish Association of Pancreatology
AFR: aggressive fluid resuscitation
AP: acute pancreatitis
ARDS: acute respiratory distress syndrome
AUGH: Alicante University General Hospital
BUN: blood urea nitrogen
DSMB: Data and Safety Monitoring Board
eCRF: electronic case report form
ICU: intensive care unit
LR: lactated Ringer solution
MFR: moderate fluid resuscitation
RCT: randomized controlled study
SIRS: systemic inflammatory response syndrome.
