# Supplementary material for: Early Weight-Based Aggressive vs. Non-Aggressive Goal-Directed Fluid Resuscitation in the Early Phase of Acute Pancreatitis: An Open-Label Multicenter Randomized Controlled Trial (The WATERFALL Trial), Design, and Rationale
Source: Front Med (Lausanne). 2020 Sep 2;7:440. doi: 10.3389/fmed.2020.00440 (PMC7492535; doi:10.3389/fmed.2020.00440)
Supplement: Supplementary file 1 [file Table_1.docx]

Supplementary material 1. List of participating collaborators.

| **Name** | **Center** | **City** | **Country** |
| --- | --- | --- | --- |
| Enrique de-Madaria | Gastroenterology Department, Alicante University General Hospital | Alicante | Spain |
| Karina Cárdenas-Jaén | Gastroenterology Department, Alicante University General Hospital | Alicante | Spain |
| Lucas Ilzarbe Sánchez | Gastroenterology Department,Del Mar Hospital | Barcelona | Spain |
| Eduardo Bajador Andreu | Gastroenterology Department, Zaragoza Miguel Servet Hospital | Zaragoza | Spain |
| Robin Rivera Irigoin | Gastroenterology Department, Marbella Costa del Sol Hospital | Málaga | Spain |
| Federico Bolado Concejo | Gastroenterology Department, Complejo Hospitalario de Navarra | Pamplona | Spain |
| Isabel Pascual Moreno | Gastroenterology Department, Valencia Clinic Hospital | Valencia | Spain |
| Ana Maria Sánchez Pardo | Gastroenterology Department, Valencia Clinic Hospital | Valencia | Spain |
| MªDolores Higón Ballester | Gastroenterology Department, Valencia La Fe Hospital | Valencia | Spain |
| Adolfo Del Val Antoñana | Gastroenterology Department, Valencia La Fe Hospital | Valencia | Spain |
| Cristina Alejandra Sánchez Gómez | Gastroenterology Department, Ourense Hospital | Ourense | Spain |
| María Francisco González | Gastroenterology Department, Ourense Hospital | Ourense | Spain |
| Raquel Fernández González | Gastroenterology Department, Ourense Hospital | Ourense | Spain |
| Coral Tejido Sandoval | Gastroenterology Department, Ourense Hospital | Ourense | Spain |
| Franco Baiocchi Ureta | Gastroenterology Department, Ourense Hospital | Ourense | Spain |
| Jose Lariño-Noia | Gastroenterology Department, Santiago Clinic Hospital | Santiago | Spain |
| MªEugenia Lauret Braña | Gastroenterology Department, Central Asturias University Hospital | Asturias | Spain |
| Manuel Alfonso Jiménez Moreno | Gastroenterology Department,Burgos University Hospital | Burgos. | Spain |
| Gadea Hontonaria Bautista | Gastroenterology Department,Burgos University Hospital | Burgos. | Spain |
| Mónica Vásquez Seoane | Gastroenterology Department,Burgos University Hospital | Burgos. | Spain |
| Alejandro Viejo-Almanzor | Gastroenterology Department, Cádiz Puerta del Mar Hospital. | Cádiz | Spain |
| Laura María Camacho Montaño | Gastroenterology Department, Cádiz Puerta del Mar Hospital. | Cádiz | Spain |
| Alba Lira Aguilar | Gastroenterology Department,Parc Taulí Consorcio Corporación Sanitaria. | Sabadell | Spain |
| Laura Llovet Soto | Gastroenterology Department ,Parc Taulí Consorcio Corporación Sanitaria. | Sabadell | Spain |
| Cristina Verdejo Gil | Gastroenterology Department, Fundación Alcorcón University Hospital. | Madrid | Spain |
| Isabel Conde Amiel | Gastroenterology Department, Valencia General Hospital. | Valencia | Spain |
| Eduardo Redondo-Cerezo | Gastroenterology Department,Virgen de las nieves University Hospital. | Granada | Spain |
| Claudia Sánchez Marín | Gastroenterology Department, Marqués de Valdecilla University Hospital. | Santander | Spain |
| Álvaro Terán Lantarón | Gastroenterology Department, Marqués de Valdecilla University Hospital. | Santander | Spain |
| María Moris Felgueroso | Gastroenterology Department, Marqués de Valdecilla University Hospital. | Santander | Spain |
| Idaira Fernandez Cabrera | Gastroenterology Department, Dr Negrín Hospital. | Las Palmas de Gran Canaria | Spain |
| Alexia Oliva García | Gastroenterology Department, Dr Negrín Hospital. | Las Palmas de Gran Canaria | Spain |
| Eva Martí Marqués | Gastroenterology Department, Lugo, University Hospital. | Lugo | Spain |
| Ana García García-de-Paredes | Gastroenterology Department, Ramón y Cajal Hospital. | Madrid | Spain |
| Javier Martínez González | Gastroenterology Department, Ramón y Cajal Hospital. | Madrid | Spain |
| Miguel Ángel Rodríguez Gandía | Gastroenterology Department, Ramón y Cajal Hospital. | Madrid | Spain |
| Rosario González Alonso | Gastroenterology Department, Ramón y Cajal Hospital. | Madrid | Spain |
| Alfonso San Juan Benito | Surgery Department, Ramón y Cajal Hospital | Madrid | Spain |
| Luís M. Oms Bernad | Gastroenterology Department,Terrassa Hospital. | Barcelona | Spain |
| Alejandro A. Giménez Villarejo | Gastroenterology Department , Clínicas Hospital | Asunción | Paraguay |
| Edgard E. Lozada Hernández | Surgery Department, Alta Especialidad del Bajío Regional Hospital | León | México |
| Rodrigo Mansilla Vivar | Gastroenterology Department, Puerto Montt Hospital | Puerto Montt | Chile |
| Rómulo Darío Vargas Rubio | Gastroenterology Department,San Ignacio, Hospital | Bogota | Colombia |
| Fabián E. Puentes | Gastroenterology Department, Unión de cirujanos, Caldas University S.A.S | Manizales | Colombia |
| Guillermo Rafael Veitia Velásquez | Gastroenterology Department, Vargas de Caracas Hospital | Caracas | Venezuela |
| Graciela Salinas Camargo | Gastroenterology Institute, Sucre | Sucre | Bolivia |
| Marco Vito Marino | Gastroenterology Department, Azienda Ospedaliera Ospedali Riuniti Villa Sofia-Cervello Hospital | Palermo | Italia |
| Jorge Alberto Navarro | Surgery Department,Mayor-Mederi Universitary Hopital | Bogota | Colombia |
| Susana Rojas López | Surgery Department,Mayor-Mederi Universitary Hopital | Bogota | Colombia |
| Paula L. Torres-Gómez | Surgery Department, Mayor-Mederi, University Hospital | Bogotá | Colombia |
| Lumír Kunovský | Gastroenterology Department, University Hospital Brno | Praga | Czech Republic |
| Pablo Salgado | Gastroenterology Department,Teodoro Maldonado Carbo | Guayaquil | Ecuador |
| Andrés Serrano | Gastroenterology Department,Teodoro Maldonado Carbo Hospital | Guayaquil | Ecuador |
